# Association between the risk of malnutrition and sarcopenia at 4.2 years of follow-up in community-dwelling older adults

**DOI:** 10.3389/fmed.2024.1363977

**Published:** 2024-02-26

**Authors:** Helen J. Vidaña-Espinoza, Miriam T. López-Teros, Julián Esparza-Romero, Oscar Rosas-Carrasco, Armando Luna-López, Heliodoro Alemán Mateo

**Affiliations:** ^1^Coordinación de Nutrición, Centro de Investigación en Alimentación y Desarrollo (CIAD), A.C., Hermosillo, Sonora, Mexico; ^2^Centro de Evaluación del Adulto Mayor, Departamento de Salud, Universidad Iberoamericana Ciudad de México, Ciudad de México, Mexico; ^3^Dirección de Investigación, Instituto Nacional de Geriatría, Ciudad de México, Mexico

**Keywords:** nutritional status, risk of malnutrition, mini-nutritional assessment, sarcopenia, fat mass, older adults

## Abstract

**Introduction:**

Currently, there is only scarce evidence of a causal association between risk of malnutrition (RM) by the mini-nutritional assessment (MNA) and the incidence of sarcopenia. This study was designed to assess such an association at 4.2 years of follow-up in community-dwelling subjects over 60 years old.

**Methods:**

The data used were from the FraDySMex cohort study. The exposition variables were RM diagnosed by the long forma of the MNA (MNA-LF) and short form (MNA-SF). The last one included the body mass index and calf circumference at baseline, while sarcopenia was diagnosed by the EWGSOP2 at follow-up and taken as the response variable. Several covariates involved in the association were also considered. A multiple logistic regression analysis was performed to test the association.

**Results:**

At baseline, 27.0 and 37.9% of subjects had RM by the MNA-LF and MNA-SF, respectively. The incidence of sarcopenia was 13.7%. The fat mass variable significantly modified the association, so it was tested in each stratum. Two independent models showed that subjects with RM by the MNA-LF in the normal fat mass stratum were at a higher risk for developing sarcopenia at follow-up than those without RM (OR 9.28; IC 95% 1.57–54.76) after adjusting for age, sex, and waist circumference. No association was found for the excess fat mass stratum subjects. Subjects with RM by the MNA-SF in the excess fat mass stratum were more likely to develop sarcopenia at follow-up than those without RM by the MNA-SF (OR 3.67; IC 95% 1.29–10.43). This association was not found in the subjects in the normal fat mass stratum.

**Conclusion:**

The association was dependent on the variable fat mass. The two forms of the MNA should not be applied indistinctly with older adults. Based on these results, it is clear that the risk of malnutrition precedes the onset of sarcopenia.

## 1 Introduction

Sarcopenia is recognized as a muscle disorder by the World Health Organization with a specific code (M62.84) from the 10th revision of the International Classification of Diseases (ICD-10) (1). According to the European Working Group on Sarcopenia in Older People (EWGSOP2) (2), sarcopenia is characterized by low muscle strength, muscle mass, and physical performance. This muscular disease increases the risk of falls (3), functional decline (4, 5), and frailty (2, 6), as well as dysphagia (7), cognitive impairment (8), and chronic diseases like hypertension (9), diabetes (10), and osteoporosis (11), nonalcoholic fatty liver disease (12), and depression (13), as well as major risk of mortality (14).

The worldwide prevalence of sarcopenia diagnosed by the EWGSOP2 (2) criteria among community-dwelling older adults is 10% (15). In Mexico, the prevalence estimated by these criteria is 14.9% in community-dwelling adults aged 50 years and over (16), but few studies on the incidence of sarcopenia have been published internationally. Estimated incidence for community-dwelling older adults in nations like Belgium and Thailand is 15 and 22%, respectively (17, 18) but no estimates are available for Mexico and other Latin American countries. Clearly, there is a need for more cohort studies that report data on the incidence of three conditions –probable, confirmed, and severe sarcopenia– as this would allow public health policies to implement timely methods to diagnose this disease and prevent its development in populations of community-dwelling older adults.

In terms of etiology, sarcopenia is recognized as a multifactorial condition. The aging process contributes to its development through diverse mechanisms, while systemic diseases as diabetes (19, 20), malignities, and organic insufficiencies may also be involved as chronic kidney disease (2, 19, 20). Recent reports suggest that alterations of people’s nutritional status due to low caloric and protein ingestion –especially malnutrition and the risk of malnutrition (RM)– are associated independently with a greater risk of developing sarcopenia in older adults (2, 17, 21–23). One systematic review analyzing only cross-sectional studies reported a significant association between malnutrition and sarcopenia in older community-dwelling adults (21). These findings have been confirmed by some cohort studies (17, 22, 23). Likewise, it has been reported that this association is independent of the diagnostic method related to the etiology of malnutrition (22).

Regarding the risk of malnutrition, some recent cross-sectional studies (24–26) and a systematic review and meta-analysis (21) analyzing cross-sectional evidence reported that older adults at risk of malnutrition, diagnosed mainly by the mini-nutritional assessment (MNA), also had a greater risk of developing sarcopenia. The only cohort study published to date showed that subjects with RM diagnosed by MNA in its two forms (MNA-SF and MNA-LF) had almost, or more than, a twofold risk of developing sarcopenia, respectively, at follow-up. However, after adjusting for several covariates in both models, that association lost statistical significance (17). Overall, the importance of this association is that RM is a highly prevalent condition in older adults worldwide (27) living in different contexts, care settings, and community (28). Mexico is no exception, as prevalences of RM as high as 50% were reported in community-dwelling older adults in a study that used non-representative sampling (29), and up to 40.4% in a representative study at the national level (30). Recently published data suggested an incidence of RM of 28.6% among community-dwelling older adults (31).

These findings of high prevalence of sarcopenia and high prevalence and incidence of RM in older adults in Mexico, coupled with the scarce, and controversial, evidence that exists worldwide on the association between RM and sarcopenia, reveal the need for more cohort studies to confirm the hypothesis of an association with RM assessed by screening tools like the MNA. The two forms of this scale are practical, economical instruments with high sensitivity and specificity for diagnosing RM among older adults (32, 33). Thus, the present study was designed to evaluate the association between the risk of malnutrition as an exposition variable and the incidence of sarcopenia as the response variable at 4.2 years of follow-up. We hypothesized that RM diagnosed by the MNA-SF and MNA-LF is causally associated with the development of sarcopenia in community-dwelling subjects over 60 years old.

## 2 Methods

### 2.1 Study design

This analysis emerged from the prospective cohort study “Fragility, Dynapenia, and Sarcopenia in Mexican Adults” (FraDySMex). Using convenience sampling, we gathered a sample of community-dwelling adults aged 50 years and over in two municipalities (out of a total of 16) of Mexico City. Participants underwent a comprehensive geriatric assessment (CGA) that included clinical, functional (physical performance and handgrip strength tests), mental, and social assessments. Body composition was assessed to diagnose osteoporosis and sarcopenia, and dynapenia and frailty, main health conditions, and their relation to various clinical outcomes. The FraDySMex study protocol conformed to the ethical principles established in the Helsinki Declaration (34), including the review of the study design by the Ethics Committee of the *Hospital General Ángeles Mocel* and the Research Committee of the *Instituto Nacional de Geriatría de México* (DI-PI-002/2014). All subjects signed their informed consent and assent to participate.

### 2.2 Recruitment of the study population

As mentioned, participants were selected by convenience sampling, so the sample may not strictly represent the community of origin. The study protocol has been published previously (35), so here we provide only a brief summary. All subjects were aged 50 years and over, and resided in the Magdalena Contreras and Álvaro Obregón sectors of Mexico City. Invitations were made through home visits by health personnel. In addition, pamphlets outlining the project were distributed in churches, community centers for older people, and social security and health centers in those areas of the city.

Potential volunteers were informed of the study protocol and invited to participate, regardless of their health, nutritional, mental, and functional status. The protocol did not consider institutionalized older adults. Eligible individuals thus included older adults who were able to move a round with or without assisting devices; could answer the study survey by themselves or helped by a caregiver and had scores of 10 points or less on the mini-mental state exam (MMSE) (36). People who presented reduced alert states due to any cause and an acute or chronic condition that, in the judgment of the medical personnel, could affect their ability to answer the survey and complete the study’s objectives, were excluded, as were those with a condition that could affect the variables of interest. Finally, volunteers who were found to have edema in the lower extremities during the medical assessment were excluded from the body composition evaluation.

### 2.3 Measurements

Baseline measurements were taken between October 2014 and December 2015 in the *Laboratorio de Investigación de Evaluación Funcional* at the *Instituto Nacional de Geriatría de México* (INGER). Follow-up was performed from October to December, 2019 in the *Centro de Evaluación del Adulto Mayor* at the *Universidad Iberoamericana* and in the INGER, in Mexico City, following the same protocol and methodologies. All procedures were conducted by a team of health professionals, previously trained and standardized.

#### 2.3.1 Baseline

Comprehensive geriatric assessment. The CGA evaluated participants’ clinical, functional, mental, and social status. In addition to providing information on their nutritional, health, and functional status, results constituted the adjustment variables used in the regression models. The CGA included an assessment of comorbidities by applying Charlson’s comorbidity scale (37). Comorbidities were defined when a subject had ≥3 diseases. Polypharmacy was determined by asking subjects to name the drugs they were taking and defined as consuming ≥5 drugs/day (38). Nutritional status was assessed by the MNA-LF and MNA-SF in its two versions with BMI and calf circumference scales which allowed us to diagnose altered nutritional states due to caloric and protein deficiencies, such as risk of malnutrition, and malnourished or malnutrition, as well as normal nutritional status (NNS). The MNA-LF and MNA-SF have sensitivities and specificities of 98 and 100%, and 96 and 98%, respectively, for diagnosing RM (32, 33). This scale was utilized following the protocol outlined in the Application Manual (www.mnaelderly.com, accessed October 1, 2014).

The CGA included an evaluation of toxicomanies. Alcohol consumption was determined by asking subjects how many alcoholic drinks they drank per day, and confirmed when the answer was ≥2 (39). Tobacco consumption was assessed by asking “do you currently smoke?,” and classified as “Yes” or “No” (40). The functional assessment covered participants’ physical self-care ability using the basic activities of daily life tool (BADL) and Barthel’s index (41). Functional dependence on the BADL was diagnosed at a score ≤ 90. Assessment of the higher-level skills that older people require to live independently in community was carried out with the instrumental activities of daily life (IADL) and Lawton & Brody’s scales (42). Functional dependence was defined by a score ≥ 1 on the IADL.

The MMSE (43) was used to assess subjects’ cognitive status and diagnose cognitive impairment, using the cut-off points for years of schooling in the Mexican population. Cognitive impairment was diagnosed when subjects with 5 or more years of schooling had MMSE scores ≤23, those with 1–4 years scored ≤19, and those with 1 or 0 years scored ≤16 (36). Depressive symptoms, from the Depression Scale of the Center for Epidemiological Studies (CESD-7) was assessed, applying the cut-off points for the Mexican population, subjects with CESD-7 scores ≥5 were classified as having depressive symptoms (44). Sociodemographic data were obtained through direct questions designed to gather information on age in years, sex as a binary variable, marital status, schooling, and living alone or accompanied.

#### 2.3.2 Other variables evaluated at baseline and incorporated into the CGA

Physical activity. It was assessed as the physical activity level (PAL), first, by estimating total energy expenditure (TEE) using a specific equation for older Hispanic adults (45), and resting metabolic rate (RMR) with an equation published previously (46). PAL was calculated using the TEE/RMR ratio and classified according to the WHO’s categories (47). Anthropometric assessment was also conducted. Body weight in kg was measured with a SECA mBCA 514 scale (MFBIA, SECA^®^, Hamburg, Germany) at a precision of 0.1 kg. Height in meters was measured with a 360 SECA 264 wireless stadiometer (SECA^®^, Hamburg, Germany) at a precision of 0.1 cm. The body mass index (BMI, kg/m^2^) was calculated. Arm, calf, and waist circumferences, and body weight and height were measured following the protocol proposed by ISAK (48). The BMI, arm and calf circumferences, in cms, were included in both forms of the MNA. Finally, we classified overweight, and obesity based on the BMI cut-off points suggested by WHO (47).

#### 2.3.3 Baseline measurements and follow-up

The variables required to diagnose sarcopenia were evaluated at baseline and follow-up, but the covariables were measured only at baseline.

Muscle strength was assessed by the handgrip strength (HGS) test using a hydraulic dynamometer (JAMAR Hydraulic, Hand Dynamometer, Lafayette, IN), following the established protocol (49). HGS in kg was stratified by sex and the BMI. To define low muscular strength, we considered the published cut-off points generated in a sample of older adults in Mexico City (16).

Appendicular skeletal muscle mass (ASM). Based on dual X-ray absorptiometry scans (DXA) (Hologic Discovery-WI, Hologic Inc., Bedford, MA, United States), appendicular lean tissue (ALT) was obtained by separating the lean mass of the arms near the thorax and of the legs near the trunk and pelvis, respecting by following the published recommendations (50). The DXA-derived ASM is considered by assuming that ALT represents mainly muscle tissue in the arms and legs, so it is the sum of limb tissue in those members. This component represents 76% of total muscle mass determined by magnetic resonance (51). Total lean tissue (TLT), fat mass (FM), total mass, and other components of body composition were obtained from the edited DXA-scans. The DXA machine was calibrated daily following the manufacturer’s instructions. The results of the ASM in kg were then divided by height^2^ in meters to obtain the appendicular skeletal muscle mass index (ASMI, kg/m^2^). Low muscle mass was diagnosed by the cut-off points derived from a sample of older adults in Mexico City (16).

Gait speed (GS). This parameter was measured using the GAIT Ride instrument (Platinum 20) (204 × 35.5 × 0.25 inches, 100 Hz sample rate) following the manufacturer’s instructions. Low gait speed was diagnosed using the cut-off points proposed by the EWGSOP2 (2).

### 2.4 Risk of malnutrition by the long and short forms of the MNA

As mentioned in the section on nutritional status, RM was assessed using the MNA-LF scale to determine three categories: NNS, 24–30 points; at risk of malnutrition, 17–23.5 points; and malnourished, <17 points. The classification based on the MNA-SF was: NNS, 12–14 points; at risk of malnutrition, 8–11 points; and malnourished, 0–7 points. Nutritional status was defined as a dichotomous variable (0 = NNS, 1 = RM). It is important to mention that none of the older adults in the sample had MNA scores <17 and ≤ 7 points at baseline, respectively, on the long and short forms. RM measured by the MNA-LF and MNA-SF were considered the hypothesis variables.

### 2.5 Sarcopenia

Sarcopenia was defined as the response variable following the EWGSOP2 criteria (2). Confirmed sarcopenia was diagnosed when a subject had low muscle strength, considering HGS adjusted for sex and the BMI (women: ≤12 kg with BMI ≤30.5 kg/m^2^ and ≤ 13 kg with BMI >30.5 kg/m^2^; men: ≤22 kg with BMI 26.6 kg/m^2^, ≤24 kg with BMI 26.7–28.5 kg/m^2^ and ≤ 22 kg with BMI >28.6 kg/m^2^) and low muscle mass, considering the ASMI (women: ≤5.35 kg/m^2^; men: ≤6.68 kg/m^2^) (16). Severe sarcopenia was diagnosed considering low strength and muscle mass plus low physical performance. In this study, we considered low physical performance as GS ≤0.8 m/s (2). Diagnoses of sarcopenia included both confirmed and severe. The latter status was diagnosed in only 7 subjects. The statistical analysis considered this a dichotomous variable (0 = without sarcopenia, 1 = with sarcopenia).

### 2.6 Covariates

Associations were recognized between diverse sociodemographic variables, such as age with sarcopenia (21, 52). The variable of age was considered continuous in years. Sex (21) was used as a categorical variable and codified as 0 = male (without risk) and 1 = feminine (with risk). In terms of health conditions, we considered comorbidity, which has been associated with sarcopenia (21), as a dichotomous variable (0 = without comorbidity, 1 = with comorbidity). Polypharmacy (53) was also taken as a dichotomous variable (0 = without polypharmacy, 1 = with polypharmacy). Cognitive impairment has been associated with sarcopenia (21, 54) and was used as a dichotomous variable (0 = without cognitive deterioration, 1 = with cognitive deterioration). Depressive symptoms also increase the risk of sarcopenia (21). This variable was codified as dichotomous (0 = without depressive symptoms, 1 = with depressive symptoms). There are also reports of a significant association of some toxicomanies, like tobacco use, with sarcopenia (21). In our analysis, tobacco consumption was used as a dichotomous variable (0 = No, 1 = Yes). Alcohol consumption has not been associated with sarcopenia, but there are reports that high consumption is related to low muscle strength and muscle mass, two components of sarcopenia (55). This was another dichotomous variable (0 = No, 1 = Yes).

Three aspects were considered covariables and incorporated into the CGA, as follows: Level of physical activity: low levels of physical activity have been associated with the development of sarcopenia (21). This was used as a dichotomous variable (0 = Active, 1 = Sedentary). Fat mass measured by DXA. Excess body fat is recognized as being associated with the development of sarcopenia or its components (56, 57). To diagnose normal and excess fat mass, we first calculated the fat mass index (FMI, kg/m^2^) based on fat mass in kg divided by height^2^ in meters, then the classification ranges of the FMI (58). This index is a gender-specific measure of fat mass not confounded by lean tissue or fat-free mass. This was used as a dichotomous variable (0 = normal fat mass, 1 = excess fat mass). Waist circumference was taken as a marker of abdominal obesity, and included as a continuous variable expressed in cms. Studies show that abdominal obesity can contribute to the development of sarcopenia due to its association with the loss of muscular strength (59).

### 2.7 Statistical analysis

To ensure data quality we first performed an exploratory analysis to observe the distribution of the variables, atypical data, means, and frequencies. Also, normality test was part of this analysis using histograms. All continues variables showed a normal distribution. We assessed significant differences in the average values or percentages of the variables between the subjects with RM and those with NNS for each form of the MNA and separately, using the Student’s *t* test and X^2^, respectively, for the continuous and categorical variables (*p* ≤ 0.05). The following formula was applied to evaluate the percentage of relative change: [(follow-up value − baseline value)/baseline value] × 100. Significant differences between the average values for age and the other anthropometric variables, body composition, and physical performance at baseline and follow-up were tested using the Student’s *t* test for paired data (*p* ≤ 0.05).

Hypothesis testing was carried out by multiple logistic regression. To generate the logistic adjustment models we began with a potential association analysis using simple logistic regression to examine the association between the response variable (sarcopenia) and the hypothesis variable (RM), and between the response variable and the other possible adjustment variables one-to-one (21, 52–54, 56, 57, 59). Potential associations were considered at a *p* value ≤0.2. The biological plausibility of associations was determined by the value of the odds ratio (OR) of each association assessed (focused on the meaning of the association as risk or protection). The variables that fulfilled both criteria were considered possible confounding variables or covariables in the multiple logistic regression analysis. The automated stepwise forward technique was utilized to generate the adjustment models.

During the automated process, the sensitivity analysis strategy was used. This mean that two possible collinear variables were not modeled together. The various preliminary adjustment models generated were assessed to identify if the adjustment variables included acted as modifier variables of the effect, or of the association between the hypothesis (RM) and response variables (sarcopenia). Modifications of the effect (interaction) were considered when the *p* value of the possible interaction variable was <0.1. We also evaluated collinearity among all the variables in the model (adjustment variables and hypothesis variable) using a correlation matrix (r), considering the absence of correlation at a negative or positive *r* value ≤0.85. The linearity assumption was tested by the ln (odds) scatters plots graph.

The final adjustment models generated by multiple logistic regression were used to test the hypothesis; that is, to assess the association between RM diagnosed by the MNA-LF and MNA-SF and the incidence of sarcopenia, considering an association of risk at a value of the OR > 1 and a *p* value ≤0.05. All analyses were carried out with the STATA 17.0 statistical package (Stata Corp, College Station).

## 3 Results

### 3.1 Characteristics of the sample at baseline

**Figure 1:**
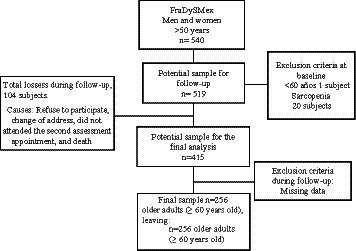
Flow chart of participants during the cohort study.

The total sample included 540 subjects aged 50 years and over. At baseline, 20 (3.7%) 14 women (70.0%) and 6 men (30.0%) met the criteria for sarcopenia. Those subjects were excluded from the follow-up cohort, so 520 men and women were free of the exposition variable and ready for the follow-up study. One volunteer was excluded due to age (<60 years), leaving 519 potential subjects for the follow-up phase. Of these individuals, 415 ≥ 60 years had complete data from the initial and final evaluations, but not all had the follow-up data required for the present analysis. As a result, 159 were excluded due to the absence of data for the variables HGS and body composition by DXA. However, a significant proportion of these subjects were not available for the follow-up study for four reasons: refusal to participate in the second phase, change of address, failure to keep appointments, and death (Figure 1). The final sample included 256 subjects, all of whom had complete measurements at baseline and follow-up. Figure 1 is a flow chart of participants during the cohort study.

The analysis of baseline data (*n* = 256) showed that the average values for HGS, ASMI, and GS were 16.9 ± 7.4 kg, 6.3 ± 0.9 kg/m^2^, and 1.0 ± 0.2 m/s, respectively, with 34.0% of subjects having low HGS, 22.3% low ASMI, and 47.6% low GS. Average age was 71.3 ± 7.3 years, 28.9% of subjects were married, 28.5% single or divorced, and 42.4% widowed. In the final sample, 42.3% had low schooling, and 24.6% reported living alone. Results showed that 14.0% had cognitive impairment, 42.9% depressive symptoms, 27.3% comorbidities, 51.1% polypharmacy, 13.6% mild dependence according to the ABVD, and 32.8% mild dependence on the AIVD. Only 8.5 and 5.8% reported tobacco and alcohol consumption, respectively. In terms of physical activity, 58.9% had low levels. Based on the BMI classification, 47.8% were overweight, and 30.9% were obese. The FMI classification found that 85 and 15%, respectively, had excess and normal fat mass. Nutritional status was evaluated by the MNA, where 27.0 and 37.9%, respectively, of subjects were found to be at risk of malnutrition, according to their MNA-LF and MNA-SF with BMI and calf circumference scores.

Table 1 shows the significant differences between the RM and NNS groups assessed by the MNA-LF and MNA-SF separately for the diverse variables. It also shows that between-group differences were dependent on the MNA form applied. Overall, significant between-group differences were found (*p* > 0.05) for the following variables: sex and schooling, comorbidity and functional dependence, alcohol consumption, and sedentarism.

**Table 1:** Characteristics of participants of the sociodemographic variables, health conditions, functionality, toxicomanies, physical activity level, anthropometry, body composition, and physical performance at baseline, according to nutritional status assessed by the two MNA forms.

	MNA-LF			MNA-SF		
Variables	RM (*n*=69)	NNS (*n*=187)	*p*-value	RM (*n*=97)	NNS (*n*=159)	*p*-value
Age, years	76.6±7.9	75.4±8.0	0.285	76.4±8.7	75.3±7.5	0.283
Gender						
Female	58 (84.0)	157 (83.9)		74 (77.1)	141 (88.7)	
Male	11 (16.0)	30 (16.1)	0.863	23 (22.9)	18 (11.3)	0.013
Marital status						
Married	23 (33.3)	52 (27.8)		34 (34.4)	41 (25.8)	
Single/Divorced	16 (23.1)	56 (29.9)		32 (33.4)	40 (25.2)	
Widowed	30 (56.5)	79 (42.2)	0.201	31 (32.2)	78 (49.0)	0.152
Education						
≤10 years	39 (56.5)	69 (36.8)		35 (36.8)	73 (45.9)	
≥11 years	30 (43.5)	118 (63.2)	0.025	62 (33.2)	86 (54.1)	0.157
Living alone						
Yes	14 (20.3)	49 (26.2)		18 (18.5)	45 (28.3)	
No	55 (79.7)	138 (73.8)	0.194	79 (81.5)	114 (71.7)	0.086
Cognitive impairment/MMSE						
Yes	15 (21.7)	21 (11.2)		13 (13.4)	23 (14.4)	
No	54 (78.3)	166 (88.8)	0.061	84 (86.6)	136 (85.6)	0.837
Depression symptoms/CESD-7						
≥5 points	37 (53.6)	73 (39.0)		35 (36.0)	75 (47.1)	
≤4 points	32 (46.4)	114 (61.0)	0.123	62 (64.0)	84 (52.9)	0.094
Comorbidity, diseases						
≥3	26 (37.6)	44 (23.5)		18 (18.5)	52 (32.7)	
≤2	43 (62.4)	143 (76.5)	0.064	79 (81.5)	107 (68.3)	0.015
Polypharmacy, drugs/day						
≥5	41 (59.4)	88 (47.0)		49 (51.0)	80 (50.3)	
≤4	28 (40.6)	99 (53.0)	0.232	48 (49.0)	79 (49.7)	0.989
ABVD dependency						
≤90 points	22 (31.8)	13 (6.9)		9 (9.2)	26 (16.4)	
≥91 points	47 (68.2)	174 (93.1)	0.000	88 (90.8)	133 (83.6)	0.116
AIVD dependency						
<1 points	34 (49.2)	50 (26.7)		17 (17.9)	67 (42.1)	
≥2 points	35 (50.8)	137 (73.3)	0.003	80 (82.1)	92 (57.9)	0.000
Smoking						
Yes	6 (8.6)	16 (8.5)		8 (8.3)	14 (8.8)	
No	63 (91.4)	171 (91.5)	0.883	89 (91.7)	145 (91.2)	0.896
Alcohol consumption						
≥3 glasses/day	5 (7.2)	9 (4.2)		9 (9.3)	5 (3.4)	
≤2 glasses/day	64 (92.8)	178 (95.8)	0.546	88 (90.7)	154 (96.6)	0.034
PAL						
Sedentary ≤1.60	53 (76.8)	98 (52.4)		62 (63.9)	89 (56.0)	
Active ≥1.61	16 (23.2)	89 (47.6)	0.005	35 (66.1)	70 (44.0)	0.175
Height, m	1.5±7.8	1.5±7.7	0.154	1.5±7.3	1.5±8.6	0.626
Body weight, kg	60.2±12.7	66.6±12.7	0.000	61.7±11.4	66.7±13.6	0.002
Total mass, kg	58.5±14.9	64.1±13.7	0.005	60.1±11.2	64.0±15.7	0.035
Total lean tissue, kg	33.6±7.5	35.4±7.3	0.078	34.2±7.2	35.4±7.5	0.246
Fat mass, kg	23.9±7.6	27.3±7.8	0.001	24.0±6.6	27.8±8.2	0.000
ASM, kg	13.4±2.3	15.6±3.4	0.006	14.1±3.0	15.2±3.3	0.046
BMC, kg	1.5±0.4	1.7±0.8	0.113	1.7±1.1	1.6±0.4	0.233
TLTI, kg	35.2±7.8	37.2±7.6	0.070	36.0±7.5	37.0±7.8	0.301
BMI, kg/m^2^	26.9±4.6	29.3±5.0	0.000	27.3±4.2	29.4±5.3	0.001
FMI, kg/m^2^	10.7±3.2	12.1±3.5	0.005	10.7±3.0	12.3±3.6	0.000
ASMI, kg/m^2^	6.0±1.6	6.4±1.1	0.056	5.0±1.0	6.5±1.4	0.026
WC, cm	89.7±11.8	94.2±13.7	0.014	90.6±9.9	94.3±14.9	0.030
MNA score	20.9±2.4	26.5±1.5	0.000	10.8±1.7	13.2±1.2	0.000
NS by BMI						
Underweight	3 (4.1)	0 (0.0)		2 (1.8)	0 (0.0)	
Normal	21 (30.1)	28 (15.9)		16 (17.0)	40 (25.0)	
Overweight	29 (42.4)	93 (50.0)		43 (44.0)	86 (54.2)	
Obese	16 (23.2)	66 (34.0)	0.000	36 (37.1)	33 (20.8)	0.369
Fat mass by FMI						
Normal	18 (26.2)	19 (10.4)		18 (18.8)	20 (12.6)	
Excess	51 (73.8)	168 (89.6)	0.001	79 (81.2)	139 (87.4)	0.180
Gait speed, m/s	0.7±0.2	0.8±0.1	0.000	0.8±0.1	0.7±0.2	0.072
HGS, kg	12.4±5.2	14.6±6.5	0.015	14.6±7.2	13.6±5.6	0.217

MNA-LF, mini-nutritional assessment, long form; MNA-SF, mini-nutritional assessment, short form; MMSE, mini-mental state examination; CESD-7, Center for Epidemiological Studies depression scale; BADL, basic activities of daily living scale; IADL, instrumental activities of daily living scale; ASM, appendicular skeletal muscle mass; TLTI, total lean tissue index; BMC, bone mineral content; BMI, body mass index; FMI, fat mass index ranges (kg/m^2^); ASMI, appendicular skeletal muscle mass index; WC, waist circumference; NS, nutritional status; FM, fat mass; HGS, handgrip strength.

The comparative analysis of the groups revealed that the average values for body weight, BMI, and waist circumference were significantly lower in the RM subjects than in the NNS group, independently of the MNA form applied. Similar findings appeared for the markers of energy reserves and body protein as for fat mass, the FMI, appendicular skeletal muscle mass, and the ASMI. Average HGS and GS values were also significantly lower in the RM group measured by the MNA-LF. The MNA scores from both forms were lower in the RM group than the NNS group, independently of the form used. In contrast, according to their MNA-LF scores, the NNS subjects had a significantly greater prevalence of overweight and obese individuals, assessed by the BMI, compared to the RM subjects. This result was also found for excess fat mass by the FMI classification. Thus, it is important to note that a significant proportion of the subjects with RM diagnosed by MNA-LF were also overweight (42.5%) or obese (23.2%) and had excess fat mass (73.8%). Similar results were found in the RM subjects assessed by the MNA-SF (Table 1).

### 3.2 Percentage of relative change at 4.2 years of follow-up

Subjects (*n* = 256, 83.9% women, 16.1% men) were followed for a period average of 4.2 years. The analysis of the average values of the baseline and follow-up measurements found a positive percentage of relative change for the variable of age (*p* = 0.001), but a negative one for all the anthropometric and body composition variables, as well as for GS and HGS in these community-dwelling older adults. No changes in the variables of height and bone mineral content were observed during the follow-up period (Table 2).

**Table 2:** Percentage of relative change in the age, anthropometry, body composition, and physical performance variables at 4.2 years of follow-up.

Variables	Baseline	Follow-up	*p*-value	∆
Age, years	71.3±7.3	75.6±7.5	0.000	5.6
Height, m	1.5±0.8	1.5±0.8	0.808	0.0
Body weight, kg	66.3±11.6	64.0±12.0	0.000	−3.4
Total mass, kg	64.8±12.6	62.3±12.0	0.000	−3.8
Total lean tissue, kg	36.2±7.3	35.5±7.2	0.017	−1.9
ASM, kg	14.9±3.2	14.5±3.1	0.000	−2.7
Total FM, kg	26.9±7.5	25.1±7.2	0.000	−6.6
BMC, kg	1.7±0.3	1.7±0.5	0.228	0.0
BMI, kg/m^2^	28.5±4.17	27.5±4.8	0.000	−3.5
FMI, kg/m^2^	11.5±3.2	10.9±3.2	0.000	−5.2
TLTI, kg/m^2^	15.5±2.1	15.2±2.4	0.043	−1.9
ASMI, kg/m^2^	6.3±0.9	6.2±0.9	0.001	−1.5
Gait speed, m/s	1.0±0.2	0.8±0.2	0.000	−20.0
HGS, kg	16.9±7.4	15.7±9.1	0.024	−7.1

∆, Percentage of relative change; ASM, Appendicular skeletal muscle mass.

FM, Fat mass; BMC, Bone mineral content; BMI, Body mass index; FMI, Fat mass index; TLTI, Total lean tissue index; ASMI, Appendicular skeletal muscle mass index; HGS, Handgrip strength.

### 3.3 Sarcopenia at 4.2 years of follow-up

Regarding the accumulated incidence of sarcopenia at 4.2 years of follow-up, 57.2, 11.36, and 2.34% of subjects developed probable, confirmed, and severe sarcopenia, respectively. Total incidence (confirmed + severe sarcopenia) was 13.7%. The men had a higher incidence than the women (28.2 vs. 11.6%) (*p* = 0.001). Table 3 shows the behavior of sarcopenia according to the exposition variable, RM, diagnosed by the two forms of the MNA at baseline. The subjects with RM diagnosed by the MNA-LF had a higher incidence of sarcopenia than the NNS group (18.8 vs. 11.8%), but this difference was not statistically significant. The subjects with RM diagnosed by the MNA-SF had a higher incidence of sarcopenia than the NNS group (21.6 vs. 8.8%) (*p* = 0.003).

**Table 3:** Accumulated incidence of sarcopenia according to nutritional status evaluated by the two MNA forms.

Variables	Incidence of sarcopenia		*p*-value
	Yes (*n*=35)	No (*n*=221)	
MNA-LF			
Risk of malnutrition	13 (18.8)	56 (81.2)	
Normal nutritional status	22 (11.8)	165 (88.2)	0.143
MNA-SF			
Risk of malnutrition	21 (21.6)	76 (78.3)	
Normal nutritional status	14 (8.8)	145 (91.1)	0.003

MNA-LF, Mini-nutritional assessment, long form; MNA-SF, Mini nutritional assessment, short form.

### 3.4 Modification of the effect of the association between RM diagnosed by the MNA and the incidence of sarcopenia at 4.2 years of follow-up

After carrying out the simple logistic regression analysis, we tested the association between RM assessed by the two forms of the MNA and the incidence of sarcopenia using a multiple logistic regression analysis after the sensitivity analysis. This generated 3 adjustment, or preliminary, models (Table 4). The preliminary adjustment models show that the association between RM by MNA-LF and the incidence of sarcopenia was not statistically significant, even after adjusting for diverse covariables (models 1, 2, 3). The same results were found for the association between the incidence of sarcopenia and RM by the MNA-SF, even after adjusting for certain covariables (models 1, 3). Model 2 shows that the subjects with RM by MNA-SF were more likely to develop sarcopenia after adjusting for the variables of age, sex, and physical activity level.

**Table 4:** Preliminary adjustment models for testing the association between RM by the MNA and sarcopenia.

	Model 1		Model 2		Model 3	
	OR (IC95%)	*p*	OR (IC95%)	*p*	OR (IC95%)	*p*
RM by MNA-LF						
No	Reference		Reference		Reference	
Yes	2.00 (0.94–4.23)	0.069	1.65 (0.74–3.66)	0.212	1.41 (0.61–3.25)	0.408
RM by MNA-SF						
No	Reference		Reference		Reference	
Yes	2.16 (0.97–4.81)	0.058	2.67 (1.27–5.63)	0.009	2.16 (0.99–4.70)	0.051

OR, odds ratio; IC95%, 95% confidence interval; RM, risk of malnutrition; MNA-LF, mini-nutritional assessment, long form; MNA-SF, mini-nutritional assessment, short form. Model 1: Adjusted for age and fat mass. Model 2: Adjusted for age, sex, and physical activity level. Model 3: Adjusted for age, sex, and waist circumference.

Each model in Table 4 evaluated whether adjustment variables like age, sex, fat mass, waist circumference, and physical activity level behaved as modifier variables of the effect or the association we hypothesized. The objective was to test interactions; that is, whether each independent variable in the models modified, or had a significant effect on, the association at a *p* value ≤0.1. Results show that the variable fat mass modified the effect of the association between RM by the MNA and the incidence of sarcopenia (*p* < 0.1), so we proceeded to evaluate the association between RM diagnosed by both forms and the incidence of sarcopenia in the two strata of the variable fat mass (normal and excess). Tables 5, 6 show the two new preliminary adjustment models generated for each hypothesis variable and stratum of this variable. These models included the same adjustment variables as the first ones, according to the automated stepwise strategy. The analysis of the new models did not find collinearity. All models satisfied the linearity assumption.

**Table 5:** Association between RM diagnosed by the MNA-LF and sarcopenia by fat mass stratum.

	Fat mass			
	Normal fat mass (*n*=38)		Excess fat mass (*n*=218)	
	OR (IC95%)	*p*-value	OR (IC95%)	*p*-value
Model 1				
No	1.00		1.00	
Yes	9.28 (1.5–54.7)	0.014	0.81 (0.2–2.4)	0.717
Model 2				
No	1.00		1.00	
Yes	10.08 (1.4–68.4)	0.018	0.81 (0.2–2.4)	0.719

OR, odds ratio; IC95%, 95% confidence interval. Model 1: Adjusted for age, sex, and waist circumference. Model 2: Adjusted for age, sex, and physical activity level.

**Table 6:** Association between RM diagnosed by the MNA-SF and sarcopenia by fat mass stratum.

	Fat mass			
	Normal fat mass (*n*=38)		Excess fat mass (*n*=218)	
	OR (IC95%)	*p*-value	OR (IC95%)	*p*-value
Model 1				
No	1.00		1.00	
Yes	0.62 (0.1–2.9)	0.556	3.67 (1.2–10.4)	0.014
Model 2				
No	1.00		1.00	
Yes	0.75 (0.1–3.3)	0.713	3.33 (1.2–9.2)	0.020

OR, odds ratio; IC95%, 95% confidence interval. Model 1: Adjusted for age, sex, and waist circumference. Model 2: Adjusted for age, sex and level of physical activity.

### 3.5 Final logistic adjustment models for testing the association between RM by the MNA and sarcopenia by the strata of the variable fat mass

Table 5 shows the final logistic adjustment models for each stratum of the modifying variable of the effect (fat mass). Final adjustment model 1 for stratum 1—the sub-sample of subjects with normal fat mass (Table 5)—showed that subjects with RM diagnosed by the MNA-LF were 9.28 times more likely to develop sarcopenia at 4.2 years of follow-up than those without RM (OR 9.28; IC 95% 1.57–54.76; *p* = 0.014). This association remained significant after adjusting for age, sex, and waist circumference. Final adjustment model 1 for the subjects in the second stratum (excess fat mass) showed that those with RM diagnosed by the MNA-LF were 19% less likely to develop sarcopenia than those with RM without sarcopenia (OR 0.81; IC 95% 0.26–2.41; *p* = 0.717). This association, however, lost significance after adjusting for age, sex, and waist circumference (Table 5).

Final adjustment model 2 for the subjects in the first stratum showed that those with RM by the MNA-LF were 10.08 times more likely to develop sarcopenia at follow-up than those without RM (OR 10.08; IC 05% 1.48–68.49; *p* = 0.018). This association remained significant after adjusting for age, sex, and physical activity level. The adjustment model for the subjects in the second stratum showed that those with RM diagnosed by the MNA-LF were 19% less likely to develop sarcopenia than those with RM by the MNA-LF (OR 0.81; IC 95% 0.26–2.47; *p* = 0.719), but this association lost significance after adjusting for age, sex, and physical activity level (Table 5).

Regarding the association between RM diagnosed by the MNA-SF and the incidence of sarcopenia, final adjustment model 1 for the first stratum showed that the subjects with RM were 38% less likely to develop sarcopenia at follow-up compared to those without RM (OR 0.62; IC 95% 0.13–2.98; *p* = 0.556), but this association was not significant after adjusting for age, sex, and waist circumference. Final adjustment model 1 for the subjects in the second stratum revealed that those with RM diagnosed by the MNA-SF were 3.67 times more likely to develop sarcopenia than those without RM (OR 3.67; IC 95% 1.29–10.43; *p* = 0.014). This association remained significant after adjusting for age, sex, and waist circumference (Table 6).

Following the aforementioned sensitivity strategy to test the association between RM by the MNA-SF and sarcopenia, final adjustment model 2 for the first stratum showed that the subjects with RD diagnosed by the MNA-SF were 38% less likely to develop sarcopenia at follow-up than those without RM (OR 0.75; IC 95% 0.17 3.33; *p* = 0.713). This association was not significant after adjusting for age, sex, and physical activity level. Final adjustment model 2 for subjects in the second stratum (excess fat mass) showed that those with RM were 3.33 times more likely to develop sarcopenia than those without RM (OR 3.33; IC 95% 1.20–9.22; *p* = 0.020). This association remained significant after adjusting for age, sex, and physical activity level (Table 6). It is important to mention that the results of the association were the same with the MNA-SF with BMI and with calf circumference.

## 4 Discussion

This study is the first to report the incidence of sarcopenia in Mexico and only the fourth in the world, applying the EWGSOP2 criteria. Results for the incidence of sarcopenia at 4.2 years of follow-up and the prevalence of RM at baseline were relatively high and very high, respectively, in this sample of older, community-dwelling adults. Results for the association between these two conditions showed that fat mass modified the effect of such association by revealing that the MNA tool for predicting sarcopenia must be used in accordance with fat mass stratum. Our search of the international literature found that these results were not reported in earlier published studies on the association tested.

With respect to incidence, results are very close to those published on older, community-dwelling adults in Belgium (15.6%) (19) but lower than those for a similar group of older adults in Thailand (22%) (18). Upon separating by sex, results were similar to those reported in a study of a randomized sample of Swiss men aged 60 years and over (60). As Table 3 shows, when the sample was classified as exposed (RM) and not exposed (NNS), based on the categorizations generated by the MNA-LF and MNA-SF, a significant proportion of subjects with RM diagnosed by both forms developed sarcopenia compared to those without RM. With respect to the exposition variable, the prevalence of RM was 27.0% by the MNA-LF and 37.9% by the MNA-SF, figures that are relatively high compared to those reported (27, 28) for older adults in several countries and for community-dwelling older Mexican adults (29, 30, 61–64). Upon considering the differences in the prevalence of RM diagnosed by the two MNA forms we recognized that the prevalence of RM diagnosed by the MNA-SF is greater than that estimated by the MNA-LF (65, 66), likely due to the respective sensitivity of those scales, as has been reported previously (33).

It is important to stress that the multiple logistic regression analysis revealed a significant association between RM diagnosed by both forms and sarcopenia in the different models. This is the first cohort study to report this finding. Our results differ from findings published previously (17) that reported an association between RM diagnosed by the MNA-LF and MNA-SF and the incidence of sarcopenia in separate models; however, that association loss significance after adjusting for some covariables. The higher prevalence of RM in our study is important, but upon adjusting for the variables or direct markers of adiposity –like fat mass– and indirect markers of central adiposity –like waist circumference– we found that they were determinants of this association. These adiposity markers were not considered in the only cohort study published to date (17). The most important factor in the hypothesis testing was the interaction analysis, which identified a modifying effect of certain variables, mainly fat mass measured by DXA. As mentioned earlier, this is the first study to report this finding (Tables 5, 6).

With respect to the association between RM by the MNA-LF and sarcopenia, both final adjustment models 1 and 2 for the stratum of normal fat mass showed a significant association at 4.2 years (Table 5). In fact, the subjects with RM in the normal fat mass stratum had lower average values for body weight, BMI, waist circumference, ASMI, and GS, were older, had fewer years of schooling, and were more sedentary than those with RM in the excess fat mass stratum. Regarding these findings and their potential contribution to the development of sarcopenia, some studies have reported that RM diagnosed by the MNA-LF can detect critical changes in body composition (62, 63) and physical performance (67), and, therefore, also in sarcopenia (68). Overall, these results indicate that RM diagnosed by the MNA-LF can be a good predictor of sarcopenia only in subjects with normal fat mass.

Turning to the association between RM diagnosed by the MNA-SF and the incidence of sarcopenia, both final adjustment models 1 and 2 showed a significant association at 4.2 years in the excess fat mass stratum (Table 6). At baseline, the subjects with RM in that stratum had significantly higher values for the variables of body weight, BMI, waist circumference, and fat mass, and were younger than those with RM in the normal fat mass stratum. The prevalences of overweight (62.8%) and obesity (26.6%) were higher than in the subjects with RM in the normal fat mass stratum, who did not show these conditions. Regarding these findings, both obesity (69) and higher fat mass indices have been associated with sarcopenia and greater decline in leg lean mass (56). In addition, abdominal obesity has been associated with loss of muscle strength in men. In the excess fat mass stratum, we found that 91.0% had central obesity according to the cut-off points for waist circumference proposed for older Mexican adults (38). These results suggest that RM diagnosed by the MNA-SF can be a predictor of sarcopenia in subjects with excess fat mass.

### 4.1 Physiopathological link between RM and sarcopenia

The association between RM and the incidence of sarcopenia is supported by the physiopathological link between these two conditions, where certain characteristics of RM (low weight, unintentional weight loss, low ingestion of macronutrients) potentialize the loss of components that lead to sarcopenia –for example, muscle strength and muscle mass– and reduce physical performance tested by diverse mechanisms (70–73). In addition, the physiopathological link between inflammation associated with aging, malnutrition, and sarcopenia (74) may be valid for the risk of malnutrition, though additional studies are needed to clarify if this link determines the association. Finally, a physiopathological link between obesity, inflammation and the development of sarcopenia cannot be ruled out. Excess adiposity is associated lineally with higher cytokine concentrations and, inversely, cytokines with muscle mass (75), and there is an association between obesity (56) and abdominal obesity with the decline in muscle strength in older adults (59) and with a greater risk of altered physical performance (76). Overall, more studies are needed to understand the relationship between adipose tissue accumulation and sarcopenia.

Other observations, however, show that people with excess fat mass are not exempt from presenting RM (77) since they may have reduced appetite associated with obesity and so potentialize RM. In this way, RM characterized by low weight, unintentional weight loss, and low caloric and protein ingestion may contribute to the development of sarcopenia. Once again, however, more research is required to support this hypothesis.

### 4.2 Strengths and limitations

One strength of this analysis is its design, which allowed us to establish a causal association between RM diagnosed by the long and short forms of the MNA as the exposition variable and sarcopenia as the response variable in a population of older Mexican adults. This analysis thus adds to the scarce evidence worldwide for this association in older community-dwelling adults. The use of multiple logistic regression to prove the assumptions of the regression, the fact that we considered most of the adjustment variables reported in the literature in relation to sarcopenia and, above all, the modifying effect found, are all important strengths of our hypothesis testing. Without question, the use of DXA, the track used to measure gait speed, and dynamometry with regional cut-off points are additional strengths.

One limitation of our analysis is that it is based on a convenience sample, so results cannot be generalized to the broader population. However, the study design and multiple logistic regression analysis allowed successful testing of the hypothesis variables. Another limitation of the cohort design was a bias due to the loss of some follow-up data. To test for this bias, we used Student’s *t* tests for independent samples for the continuous variables, and X^2^ for the categorical variables to detect differences at baseline between the group of losses at follow-up and the subjects who completed the study. Results showed that the average values or percentages of the diverse variables analyzed were similar between the groups, thus confirming the absence of bias. As a result, there was no concern related to a possible differentiating effect between the subjects who stayed and those who abandoned the study.

Another limitation is that we did not measure some variables at baseline that may contribute to the development of sarcopenia, such as proinflammatory markers. However, there is evidence that malnutrition diagnosed by the ESPEN criteria predict the development of sarcopenia (22) although they do not contemplate inflammation. The MNA does not consider inflammation either, so this association may be valid without adjusting for proinflammatory markers, though we recommend that future studies evaluate these markers at baseline. We contemplated the markers of adiposity as adjustment variables since the greater the amount of adipose tissue the greater the proinflammatory markers (75). Hence, we considered inflammation, though only indirectly.

## 5 Conclusion and clinical implications

Findings show that the incidence of sarcopenia is relatively high in older community-dwelling adults. The risk of malnutrition diagnosed by both forms of the MNA was associated with a greater likelihood of developing sarcopenia at 4.2 years of follow-up, considering the modifying effect of fat mass and other adjustment variables on this association. Therefore, RM diagnosed by the MNA-LF can be used with individuals with normal fat mass, while RM diagnosed by the MNA-SF can be used with people who have excess fat mass, as long as the aim is to confirm MNA as a predictor of the incidence of sarcopenia. Before applying this instrument, however, it is advisable to estimate fat mass with any predictive model published in the study region that is exact and precise for older adults (78, 79). Once determined, fat mass should be divided by height^2^ in meters to obtain the FMI and classify subjects using the cut-off points cited in the methodology section for subjects with normal and excess fat mass. Finally, our work found that the risk of malnutrition precedes the development of sarcopenia, not vice versa. However, once again, more research is required to confirm or reject the hypothesis tested using the same methodologies and procedures reported herein.

## Data availability statement

The raw data supporting the conclusions of this article will be made available by the authors, without undue reservation.

## Ethics statement

The studies involving humans were approved by the Ethics Committee of the Hospital General Ángeles Mocel and the Research Committee of the Instituto Nacional de Geriatría de México (DI-PI-002/2014). The studies were conducted in accordance with the local legislation and institutional requirements. The participants provided their written informed consent to participate in this study.

## Author contributions

HV-E: Formal analysis, Writing – original draft, Writing – review & editing. ML-T: Data curation, Funding acquisition, Methodology, Supervision, Writing – original draft, Writing – review & editing. JE-R: Formal analysis, Writing – review & editing. OR-C: Funding acquisition, Project administration, Resources, Writing – review & editing. AL-L: Writing – review & editing. HM: Conceptualization, Formal analysis, Writing – original draft, Writing – review & editing.
